# Strategies and Practices in Off-Label Marketing of Pharmaceuticals: A Retrospective Analysis of Whistleblower Complaints

**DOI:** 10.1371/journal.pmed.1000431

**Published:** 2011-04-05

**Authors:** Aaron S. Kesselheim, Michelle M. Mello, David M. Studdert

**Affiliations:** 1 Division of Pharmacoepidemiology and Pharmacoeconomics, Department of Medicine, Brigham and Women's Hospital, Boston, Massachusetts, United States of America; 2 Harvard Medical School, Boston, Massachusetts, United States of America; 3 Department of Health Policy and Management, Harvard School of Public Health, Boston, Massachusetts, United States of America; 4 School of Law, University of Melbourne, Melbourne, Australia; 5 School of Population Health, University of Melbourne, Melbourne, Australia; Yale University School of Medicine, United States of America

## Abstract

**Background:**

Despite regulatory restrictions, off-label marketing of pharmaceutical products has been common in the US. However, the scope of off-label marketing remains poorly characterized. We developed a typology for the strategies and practices that constitute off-label marketing.

**Methods and Findings:**

We obtained unsealed whistleblower complaints against pharmaceutical companies filed in US federal fraud cases that contained allegations of off-label marketing (January 1996–October 2010) and conducted structured reviews of them. We coded and analyzed the strategic goals of each off-label marketing scheme and the practices used to achieve those goals, as reported by the whistleblowers. We identified 41 complaints arising from 18 unique cases for our analytic sample (leading to US$7.9 billion in recoveries). The off-label marketing schemes described in the complaints had three non–mutually exclusive goals: expansions to unapproved diseases (35/41, 85%), unapproved disease subtypes (22/41, 54%), and unapproved drug doses (14/41, 34%). Manufacturers were alleged to have pursued these goals using four non–mutually exclusive types of marketing practices: prescriber-related (41/41, 100%), business-related (37/41, 90%), payer-related (23/41, 56%), and consumer-related (18/41, 44%). Prescriber-related practices, the centerpiece of company strategies, included self-serving presentations of the literature (31/41, 76%), free samples (8/41, 20%), direct financial incentives to physicians (35/41, 85%), and teaching (22/41, 54%) and research activities (8/41, 20%).

**Conclusions:**

Off-label marketing practices appear to extend to many areas of the health care system. Unfortunately, the most common alleged off-label marketing practices also appear to be the most difficult to control through external regulatory approaches.

*Please see later in the article for the Editors' Summary*

## Abstract 2

Aaron Kesselheim and colleagues analyzed unsealed whistleblower complaints against pharmaceutical companies filed in US federal fraud cases that contained allegations of off-label marketing, and develop a taxonomy of the various off-label practices.

## Editors' Summary

**Background:**

Before a pharmaceutical company can market a new prescription drug in the US, the drug has to go through a long approval process. After extensive studies in the laboratory and in animals, the pharmaceutical company must test the drug's safety and efficacy in a series of clinical trials in which groups of patients with specific diseases are given the drug according to strict protocols. The results of these trials are reviewed by Federal Drug Administration (FDA, the body that regulates drugs in the US) and, when the FDA is satisfied that the drug is safe and effective for the conditions in which it is tested, it approves the drug for sale. An important part of the approval process is the creation of the “drug label,” a detailed report that specifies the exact diseases and patient groups in which the drug can be used and the approved doses of the drug.

**Why Was This Study Done?:**

Physicians can, however, legally use FDA-approved drugs “off-label.” That is, they can prescribe drugs for a different disease, in a different group of patients, or at a different dose to that specified in the drug's label. However, because drugs' manufacturers stand to benefit financially from off-label use through increased drugs sales, the FDA prohibits them from directly promoting unapproved uses. The fear is that such marketing would encourage the widespread use of drugs in settings where their efficacy and safety has not been rigorously tested, exposing patients to uncertain benefits and possible adverse effects. Despite the regulatory restrictions, off-label marketing seems to be common. In 2010, for example, at least six pharmaceutical companies settled US government investigations into alleged off-label marketing programs. Unfortunately, the tactics used by pharmaceutical companies for off-label marketing have been poorly understood in the medical community, in part because pharmaceutical industry insiders (“whistleblowers”) are the only ones who can present in-depth knowledge of these tactics. In recent years, as more whistleblowers have come forward to allege off-label marketing, developing a more complete picture of the practice is now possible. In this study, the researchers attempt to systematically classify the strategies and practices used in off-labeling marketing by examining complaints filed by whistleblowers in federal enforcement actions where off-label marketing by pharmaceutical companies has been alleged.

**What Did the Researchers Do and Find?:**

In their analysis of 41 whistleblower complaints relating to 18 alleged cases of off-label marketing in federal fraud cases unsealed between January 1996 and October 2010, the researchers identified three non–mutually exclusive goals of off-label marketing schemes. The commonest goal (85% of cases) was expansion of drug use to unapproved diseases (for example, gabapentin, which is approved for the treatment of specific types of epilepsy, was allegedly promoted as a therapy for patients with psychiatric diseases such as depression). The other goals were expansion to unapproved disease subtypes (for example, some antidepressant drugs approved for adults were allegedly promoted to pediatricians for use in children) and expansion to unapproved drug dosing strategies, typically higher doses. The researchers also identified four non–mutually exclusive types of marketing practices designed to achieve these goals. All of the whistleblowers alleged prescriber-related practices (including providing financial incentives and free samples to physicians), and most alleged internal practices intended to bolster off-label marketing, such as sales quotas that could only be met if the manufacturer's sales representatives promoted off-label drug use. Payer-related practices (for example, discussions with prescribers about ways to ensure insurance reimbursement for off-label prescriptions) and consumer-related practices (most commonly, the review of confidential patient charts to identify consumers who could be off-label users) were also alleged.

**What Do These Findings Mean?:**

These findings suggest that off-labeling marketing practices extend to many parts of the health care delivery system. Because these practices were alleged by whistleblowers and were not the subject of testimony in a full trial, some of the practices identified by the researchers were not confirmed. Conversely, because most of the whistleblowers were US-based sales representatives, there may be other goals and strategies that this study has not identified. Nevertheless, these findings provide a useful snapshot of off-label marketing strategies and practices allegedly employed in the US over the past 15 years, which can now be used to develop new regulatory strategies aimed at effective oversight of off-label marketing. Importantly, however, these findings suggest that no regulatory strategy will be complete and effective unless physicians themselves fully understand the range of off-label marketing practices and their consequences for public health and act as a bulwark against continued efforts to engage in off-label promotion.

**Additional Information:**

Please access these Web sites via the online version of this summary at http://dx.doi.org/10.1371/journal.pmed.1000431.

- The US Food and Drug Administration provides detailed information about drug approval in the US for consumers and for health professionals; its Bad Ad Program aims to educate health care providers about the role they can play in ensuring that prescription drug advertising and promotion is truthful and not misleading.
- The American Cancer Society has a page about off-label drug use
- Wikipedia has pages on prescription drugs, on pharmaceutical marketing, and on off-label drug use (note that Wikipedia is a free online encyclopedia that anyone can edit; available in several languages)
- Taxpayers Against Fraud is a nonprofit organization dedicated to helping whistleblowers, and it presents up-to-date information about False Claims Act cases
- The Government Accountability Project is a nonprofit organization that seeks to promote corporate and government accountability by protecting whistleblowers, advancing occupational free speech, and empowering citizen activists
- Healthy Skepticism is an international nonprofit membership association that aims to improve health by reducing harm from misleading health information

## Introduction

In the US, a setting dominated by aggressive advertising of prescription drugs to patients and physicians, off-label marketing has been a controversial subject area. Physicians are permitted to prescribe drugs “off label”—that is, for purposes and patient populations outside of those formally approved by the US Food and Drug Administration (FDA). However, the FDA prohibits pharmaceutical companies from engaging in direct promotion of those unapproved uses [1]. The rationale is that such marketing can lead to widespread uses of a drug that are not based on evidence of efficacy and safety, expose patients to uncertain benefits and the prospect of adverse effects, and undermine incentives for manufacturers to conduct clinical trials necessary to achieve FDA approval for new uses [2]–[5].

Despite regulatory restrictions on off-label marketing, the practice appears to have flourished [6],[7]. In 2009, Pfizer paid US$2.3 billion to settle allegations that it marketed its drugs illegally to physicians—the largest federal health care fraud settlement in US history [8]. In 2010, at least six other manufacturers settled charges pertaining to off-label marketing, and more were under investigation [9]–[15]. The widely publicized litigation over the anti-inflammatory drug rofecoxib (Vioxx) also exposed marketing practices, such as seeding trials and ghost-writing of medical journal articles, that could promote off-label uses [16],[17]. What is known about off-label marketing practices comes largely in this form—namely, episodic reporting of high-profile prosecutions in the popular media [18]–[20], or personal testimony or congressional investigations arising from these same cases [21],[22]. There has been no systematic collection and analysis of these cases, which makes it difficult to identify larger themes and draw conclusions about the favored tactics.

An accumulating number of these cases over the last decade makes such an analysis feasible. Moreover, the data available to conduct this type of analysis are remarkably rich because virtually all of the major cases have been instigated by “whistleblowers” whose complaints provide detailed, firsthand knowledge of the practices at issue [23]. Because off-label marketing activities are secretive and difficult to detect and examine through other means [24], reports from these insiders provide a uniquely illuminating perspective on the range and nature of practices pursued.

We analyzed whistleblower-initiated legal complaints filed in off-label marketing cases over the last 15 y to shed more light on this widely discussed but poorly understood challenge for health regulation. We aimed to create a typology for understanding these cases and a coherent thematic model for mapping pharmaceutical companies' fraudulent promotional behaviors and strategies. Improved understanding in this area has the potential to contribute to the development of strategies for better detection and enforcement.

## Methods

### Design Overview

The primary data for this study consisted of complaints filed by whistleblowers in “qui tam” cases brought under the US federal False Claims Act (FCA). In brief, the FCA prohibits the submission of false claims to the government for reimbursement. Private citizens who notice potential violations of the FCA can file a sealed complaint in federal court; those who do nearly always retain a personal attorney to represent them and help them write their complaint. The allegations in the complaint are then investigated by the US Department of Justice (DOJ)-Civil Division, which, depending on the strength of the evidence, may elect to intervene and take over the enforcement action, essentially inserting the government as the lead party in the case. At this point, the original whistleblower's complaint is usually unsealed. Multiple complaints may be filed against the same company, but the DOJ intervenes only on the first complaint brought to its attention or subsequent complaints that provide new information (other nonintervened complaints against the same company are usually dismissed and remain sealed). Because of this screening process and the clout of the DOJ, nearly all complaints in which the DOJ intervenes lead to a settlement or judgment against the defendant company. This study focused on cases against pharmaceutical manufacturers for off-label marketing of prescription drugs in which the DOJ intervened.

### Setting and Participants

Officials in the DOJ-Civil Division provided us with a full list of pharmaceutical-related federal qui tam cases in which the DOJ intervened and that were settled between January 1996 and 2005. We updated the list to include all DOJ-intervened cases through October 2010 by conducting a search of DOJ press releases [25] and electronic media reports in Lexis-Nexis. We cross-checked the final list with data compiled by Taxpayers Against Fraud, a nongovernmental organization that tracks federal fraud actions. We then obtained the unsealed complaints in these cases from the DOJ, on-line searches of archives of US federal court filings [26], and direct approaches to lawyers involved in the litigation.

Complaints are written documents that generally consist of a summary of the allegations, a description of the whistleblower(s) and defendants, and a detailed account of the allegations and the evidence supporting them. They may be amended during the course of the investigation. We used the most recent versions of the whistleblower-filed complaints available and accessible at the extraction date (6 November 2010). We searched the summaries of the allegations to determine which made allegations about unlawful off-label marketing by the defendant company; 41 complaints in 18 cases did. These complaints formed our analytical sample. Copies of the complaints can be found at http://www.drugepi.org/education/primarydocs.php.

### Qualitative Analysis

We designed a structured instrument for abstracting information from the complaints. An initial typology was generated using a standard coding methodology [27],[28]. Two investigators (ASK and DMS) acting independently conducted a preliminary review of 20% of the complaints. After comparing and discussing results of these reviews, we identified two major descriptive domains for further analysis: the strategic goal of the off-label marketing scheme and the specific practices manufacturers used to achieve that goal. We also identified categories and subcategories within each of those domains. One of us (ASK) then read each complaint and coded the details provided into the prespecified categories and subcategories in each domain.

It is important to note that the range of off-label marketing strategies and behaviors we identified and report below are drawn from across the sample of cases as a whole; no manufacturer was accused of all of them.

## Results

A total of 41 complaints arose from 55 whistleblowers (Table 1). At the time of the alleged fraud, the whistleblowers worked as pharmaceutical sales representatives (39/55, 71%), sales or accounting managers (11/55, 20%), and unaffiliated physicians (5/55, 9%). The cases were brought against 18 manufacturers, including both large companies with diverse drug portfolios (e.g., Pfizer, Eli Lilly) and smaller companies selling a relatively narrow range of products (e.g., Orphan Medical, Medicis). At the time of analysis, settlements had occurred in 16 of the 18 cases and totaled US$7.9 billion in damages.

**Table 1:** Pharmaceutical fraud cases related to off-label marketing, January 1996–October 2010.

Company Name	Complaints	Complainants	Drug(s)	Case Status
Parke-Davis/Warner-Lambert	1	Medical marketing liaison	Gabapentin (Neurontin)	Settled in 2004 (US$430 million)
Serono	3	Sales representatives, marketing managers, unaffiliated nonprofit organization	Somatropin (Serostim)	Settled in 2005 (US$704 million)
InterMune	1	Sales representative	Interferon gamma 1b (Actimmune)	Settled in 2006 (US$37 million)
Bristol Myers-Squibb	1	Business manager	Pravastatin (Pravachol), Metformin (Glucophage), others	Settled in 2007 (US$515 million)
Cell Therapeutics	1	Sales representative	Arsenic trioxide (Trisenox)	Settled in 2007 (US$11 million)
Orphan Medical	1	Sales representative	Sodium oxybate (Xyrem)	Settled in 2007 (US$20 million)
Medicis	1	Sales representatives	Ciclopirox gel (Loprox)	Settled in 2007 (US$10 million)
Cephalon	4	Sales representatives, sales manager, unaffiliated physician	Modafinil (Provigil), Tiagabine (Gabitril), Fentanyl buccal (Actiq)	Settled in 2008 (US$425 million)
Eli Lilly	4	Sales representatives	Olanzapine (Zyprexa), others	Settled in 2009 (US$1.4 billion)
Pfizer	8	Sales representatives, sales managers, unaffiliated physician	Valdecoxib (Bextra), Ziprasidone (Geodon), Pregabalin (Lyrica), others	Settled in 2009 (US$2.3 billion)
AstraZeneca	1	Sales representative	Quetiapine (Seroquel)	Settled in 2010 (US$520 million)
Ortho-McNeil-Janssen	2	Sales representatives, unaffiliated physician	Topiramate (Topamax)	Settled in 2010 (US$81 million)
Novartis	1	Marketing managers	Tobramycin (TOBI)	Settled in 2010 (US$72.5 million)
Forest	2	Sales representative, unaffiliated physician	Citalopram (Celexa) and Escitalopram (Lexapro)	Settled in 2010 (US$313 million)
Allergan	3	Sales representative, managers, unaffiliated physician	OnabotulinumtoxinA (Botox)	Settled in 2010 (US$600 million)
Novartis	4	Sales representatives	Oxcarbazepine (Trileptal), others	Settled in 2010 (US$422.5 million)
Scios	2	Sales directors	Nesiritide (Natrecor)	Complaints unsealed (2009)
Wyeth	1	Sales representatives	Sirolimus (Rapamune)	Complaint unsealed (2010)
****	**41**			**US$7.9 billion**

### Off-Label Marketing Strategies

According to the complaints, manufacturers aimed to increase use of their products through off-label marketing schemes in three non–mutually exclusive ways. They sought to expand uses to different disease entities, to variations on the approved indication, and to alternatives to the approved dosing schedule (Table 2).

**Table 2:** Frequency of off-label marketing strategies and practices reported in whistleblower complaints.

Descriptor	*n*/*N*, Percent
***Off-label marketing strategies***	
**Expansion to different disease entity**	**35/41, 85%**
Similar symptoms, different disease	17/35, 49%
**Expansion to variation of approved indication**	**22/41, 54%**
Different patient subgroup	10/22, 45%
**Expansion to variation of approved dosing schedule**	**14/41, 34%**
***Off-label marketing practices***	
**Prescriber-related**	**41/41, 100%**
Direct financial incentives	35/41, 85%
Distorted presentation of supporting evidence	31/41, 76%
Influence on continuing medical education programs	22/41, 54%
Influence on peer-reviewed literature, including ghost-writing	20/41, 49%
Recruitment as clinical trial investigators	8/41, 20%
Free samples	8/41, 20%
**Internal practices**	**37/41, 90%**
Intramural meetings	27/37, 73%
Internal documents, brochures	17/37, 46%
Use of company-based physicians and scientists	19/37, 51%
Cloaking strategies	25/37, 68%
Sham warnings from legal counsel	16/25, 64%
Direct orders to conceal activities	12/25, 48%
Financial incentives to employees	15/37, 41%
**Payer-related**	**23/41, 56%**
Discussions with prescribers about how to ensure reimbursement	18/23, 78%
Development of billing systems that circumvent restrictions	13/18, 72%
Falsification of billing codes	11/18, 61%
Direct approaches to payers to ensure presence on formulary	8/23, 35%
**Consumer-related**	**18/41, 44%**
Direct identification of/approaches to consumers through physician office or pharmacy	10/18, 56%
Funding of consumer organizations	3/18, 17%

#### Expansion to unapproved disease entities

The most prevalent strategy involved expanding use on the basis of diagnosis—that is, seeking off-label uses for disease entities distinct from those approved by the FDA (35/41, 85%). For example, gabapentin (Neurontin), approved as adjunctive treatment for certain types of epilepsy, was also allegedly promoted as therapy for patients with psychiatric disease such as bipolar disorder or depression [29]. Another case involved Pfizer's alleged promotion of sildenafil (Viagra) to treat low libido and to “restore and increase orgasmic sensations” in women [30].

In some cases, a reported rationale for pursuing this type of expansion was that limiting sales to the FDA-approved indication could not sustain needed levels of revenue. One whistleblower from a small, single drug-focused company stated that she was told “that management wanted to sell the company, and that in order to make it a more attractive acquisition target, it was necessary to show increased sales revenue” [31].

In many examples of this marketing strategy, the drug was promoted for treatment of similar symptoms across disease classes (17/35, 49%). For example, modafinil (Provigil), initially approved for narcolepsy-related sleepiness, was allegedly promoted for many types of sleepiness in non-narcoleptic patients [32]. Another example related to the anti-inflammatory drug valdecoxib (Bextra), which was approved for a limited number of pain-related indications and then allegedly promoted by Pfizer for pain relief more broadly [33].

#### Expansion to unapproved indications

The second most common strategy for off-label promotion was to expand the product's use to different variations of the same condition (22/41, 54%). In some cases, the off-label disease was closely related to the approved one—for example, when a product was specifically approved for a severe manifestation of a condition but then promoted for milder forms. In the case of nesiritide (Natrecor), the drug was approved for “acutely decompensated heart failure” and was allegedly promoted in patients with chronic stable heart failure as a preventative measure [34]. Although both groups of patients had heart failure, they were quite different manifestations of the disease.

One prominent subcategory of this type of off-label promotion focused on patient subgroups different from those contemplated in the FDA approval (10/22, 45%). For example, ciclopirox gel (Loprox) was approved for fungal dermatoses in patients over age 10, but allegedly promoted by its manufacturer to manage diaper-related fungal dermatitis in babies [35]. In some of the antidepressant drugs in our sample, the product was approved for adult use, but allegedly promoted to pediatricians and family practice physicians specifically for young patients who demonstrated signs of depression [30],[36]. In the case of citalopram (Celexa), studies that had shown dangers with using the drug in pediatric populations were allegedly withheld from physicians as part of the marketing campaign [36].

#### Expansion to unapproved dosing strategies

The final, and least common, variety of off-label expansion was off-label prescribing based on different dosing regimens than that approved by the FDA (14/41, 34%). Typically, manufacturers promoted higher doses to enhance revenues by encouraging sale of more units of the product. For example, the manufacturer of oxcarbazepine (Trileptal) allegedly promoted use of the antiepileptic drug “as monotherapy for seizures using extremely high dosages” [37]. By contrast, the manufacturer of sirolimus (Rapamune), which was approved for transplant patients in combination with cyclosporine and corticosteroids, allegedly trained its staff to encourage its use in combination with “any drug or combination of drugs that a physician could be convinced to prescribe” to enhance its market possibilities [38].

### Off-Label Marketing Practices

The marketing practices manufacturers allegedly employed to achieve these strategic goals for off-label use fell into four non–mutually exclusive categories: internal practices, payer-related practices, prescriber-related practices, and consumer-related practices. We defined internal practices as incentives and other aspects of the employment environment at the defendant manufacturer that encouraged employees to promote off-label uses. Payer-related practices were strategies aimed at encouraging insurers to pay for off-label prescriptions. Prescriber-related and consumer-related practices involved direct promotion of off-label drug use to prescription writers and consumers, respectively.

#### Prescriber-related practices

All of the complaints we analyzed detailed off-label promotion to prescribers; this was generally the centerpiece of the whistleblowers' complaints. Though manufacturers are not supposed to discuss off-label uses unless a physician inquires, many were accused of either flouting that rule or designing their representatives' presentations in such a way as to guarantee that discussion would inevitably lead to off-label uses.

According to the complaints, off-label use was frequently encouraged through self-serving presentations of the scientific literature through which physicians were given false or unbalanced study data supporting the unapproved use (31/41, 76%). A common example was selective presentation of favorable studies, where dangers from the off-label uses allegedly being promoted were not mentioned [39]. Other examples included presenting one drug as being superior to another when no head-to-head studies had been conducted [40] and characterizing reports of individual cases or poorly designed studies as definitive evidence supporting an off-label use [41].

A number of whistleblowers alleged that free samples had been provided (8/41, 20%) as a way to promote off-label use. The whistleblowers in this group reported that these samples were intended to encourage physicians to use a product on the basis of convenience, even though it might not be approved for a certain use. In addition, many described how free samples were intended to introduce unapproved patient populations to the manufacturer's product with the intention of stimulating their continued use.

Complaints alleged that manufacturers also encouraged off-label use through direct financial incentives to physicians. Lavish gifts or honoraria were mentioned in most complaints (35/41, 85%), with many whistleblowers reporting strategies to target these gifts to physicians who were high off-label prescribers (18/41, 44%). In some cases, physicians might be invited to serve in focus groups or as consultants to the manufacturer, although it was alleged that the association was intended not to obtain expert advice, but to provide money to prescribers to positively reinforce off-label use (15/41, 37%).

Finally, off-label use was encouraged among prescribers through teaching and research activities. In over half the cases, Continuing Medical Education (CME) seminars were organized with speakers known to promote off-label uses (22/41, 54%). In a few cases, whistleblowers reported that CME activities were organized by shell corporations to impart an appearance of scientific neutrality [34]. Nearly half of whistleblowers also alleged that manufacturers sought to promote off-label drug use through journal publications (20/41, 49%). These practices included falsely reporting outcomes from patients in manufacturer-sponsored studies [42] and publishing “ghostwritten” articles supporting an unapproved use written by the manufacturer under the name of a respected scientist [43]. Finally, a minority of whistleblowers alleged that manufacturers recruited physicians to conduct clinical trials for them with the intent of encouraging off-label use (“seeding trials”), rather than for any useful scientific or information-gathering reasons (8/41, 20%).

#### Internal practices

Thirty-seven of the whistleblower (90%) complaints detailed particular internal manufacturer practices intended to bolster the off-label marketing (two of the four complaints where these were not mentioned were filed solely by whistleblowers positioned outside the companies). All of the practices described were reported to be company-wide, rather than the work of an individual manager or group of managers. In 73% (27/37) of these cases, the off-label marketing strategy was implemented through intramural meetings and seminars in which marketing practices were discussed; in 46% (17/37) of them, it was also implemented through development of brochures and other materials for dissemination; in 51% (19/37), employees other than the sales representatives, such as internal physicians and scientists, were involved.

Many of the complaints describing internal practices (25/37, 68%) pointed to specific efforts by drug manufacturers to conceal off-label marketing activities. Some described warnings from legal teams to avoid off-label marketing (16/25, 64%). These were generally understood by employees as providing “plausible deniability” to the company [33], and were widely undermined through strategies such as verbal orders diverging from what was declared in their company policies [31]. For example, one whistleblower reported that his company purposefully designed “do not detail” labels on materials related to off-label uses that could easily be removed by a sales representative [30]. A third of complaints included reports of direct orders to conceal, such as “cleaning” internal reports and memoranda of all mentions of off-label marketing (12/25, 48%).

The complaints frequently described use of financial incentives for employees to engage in off-label marketing. Forty-one percent (15/37) of the reports of internal strategies described incentives or other aspects of employees' compensation plans that were directly tied to effectively implementing an off-label prescription strategy. In one case involving a drug approved by the FDA for a rare indication, a whistleblower reported that the company imposed sales quotas on representatives that could only be met through expanding use beyond the limited approved indication [31]. Other examples included an internal sales “contest” for employees who could demonstrate greatest compliance with marketing programs encouraging off-label use [44] and direct payments to employees to encourage them not to report off-label marketing practices [35].

#### Payer-related practices

Payer-related promotional practices were reported in just over half of the complaints (23/41, 56%) and fell into two categories: discussions with prescribers about ways to ensure insurance reimbursement for their off-label prescriptions (18/23, 78%) and direct discussions with payers themselves (8/23, 35%) (three complaints described both). The reports of discussions with prescribers in complaints described efforts to educate them about how to manage the billing system to ensure that off-label prescriptions were reimbursed, including advice on ways to bypass insurers' restrictions on prescriptions of the product (13/18, 72%). For example, one whistleblower reported being taught to overcome a requirement that patients receive a trial of a competitor's drug first by instructing physicians to issue two different prescriptions at the same time: one for the competitor's drug that the patient could ignore, the other for the company's drug [45]. The other strategy commonly reported was to encourage providers to falsify billing codes (11/18, 61%).

Seven complaints reported that manufacturers interacted with payers to encourage off-label drug use by ensuring drugs were on a formulary for off-label uses (four reports) or developing organizational protocols that included the off-label use (four reports; one reported both). One whistleblower described a bolder tactic for ensuring formulary coverage for off-label use of a product: directing “their sales representatives to garnish physician and patient letters of support to encourage reimbursement” by Medicare intermediaries [46].

#### Consumer-related practices

Nearly half the complaints described off-label marketing practices focused directly on consumers (18/41, 44%). The most common example involved identifying consumers who could be off-label users (10/18, 56%)—for instance, by conducting chart reviews in physicians' offices. The next step was bringing those patients eligible for an off-label use to the physician's attention, thereby fusing a consumer-focused practice with a prescriber-focused one. Other practices intended to directly encourage off-label use among consumers allegedly included promotion of consumer demand for off-label uses through payments to nonprofit, consumer-focused disease management organizations in exchange for their support of the off-label use [43]. Another complaint described on-line resources presented by a “noncommercial public interest organization” that were intended to promote off-label use of the product, but which were developed by a marketing firm linked to the defendant company [47]. In a third case, the whistleblowers alleged that the company provided indigent patients with “gift certificates, phone cards, and bus tokens” as inducements to seek out prescriptions of a drug for an off-label purpose [48].

## Discussion

Through a comprehensive review of whistleblower complaints, to our knowledge the first of its kind, we found descriptions of a range of marketing practices related to off-label promotion of prescription drugs. All of the strategies and behaviors we outlined were alleged by whistleblowers with special knowledge of company practices, although none of the complaints was subject to full trial and evaluation by a judge or jury. The study provides a basic empirical snapshot of the extent to which each of these strategies and practices have been employed, at least among cases exposed in qui tam litigation.

Our findings show that off-label marketing practices have a broad reach. Similar behaviors and strategies were linked to manufacturers of varying sizes across drugs in virtually all therapeutic classes; they extended to many aspects of the health care system; they affected a multitude of players (prescribers, pharmacies, disease advocacy groups, CME organizations, consumers); and were pursued through virtually every facet of physician-industry relationships (paid consultancies, preceptorships, and collaboration in clinical trials and research publications). The alleged tactics in our analytic sample ranged from subtly encouraging physicians to ask for information about off-label uses to providing strong financial rewards for encouraging off-label uses; they also included targeting multiple links in the prescription production chain, from company scientists and sales representatives to prescribers.

Some of the practices we identified have been highlighted in anecdotal reports and are relatively well known. Others have received little or no attention, such as pharmaceutical marketing representatives working directly with physicians and their office managers to circumvent reimbursement restrictions set by government payers and other insurers. Nearly a quarter of the whistleblowers alleged that pharmaceutical sales representatives were given access to patients' confidential medical records at physicians' offices for the purposes of trolling for prospective targets for illegal direct-to-consumer promotion of off-label uses. Despite the remarkable prevalence of this practice among the complaints we analyzed, media coverage has tended to center on other, more institutionally focused aspects of fraud.

New regulatory strategies, both public and private, aimed in part at preventing off-label marketing, have proliferated in recent years. Medical journals have changed their authorship standards to foil ghostwriting [49]; following the example of several states, the federal health care reform legislation requires disclosure of pharmaceutical industry payments to physicians [50]; the leading pharmaceutical manufacturers' association, PhRMA, has adopted a Code of Ethics that prohibits certain types of gifts [51]; and a handful of academic medical centers have restricted or prohibited visits by pharmaceutical sales representatives [52]. Our findings support the need for these measures to combat gifts to physicians, which we identified as the single most prevalent modality of off-label promotion reported by whistleblowers.

However, our results also suggest that additional steps will likely be necessary to curb off-label marketing. For example, interventions seeking to insulate physician education from industry influence have largely been limited to programs in which the manufacturer controls the content, but the reports in this study suggest that even so-called “unrestricted” educational grants from industry may be deployed to effect off-label marketing. A better policy solution would be fully independent programs of continuing medical education, an approach that has received limited support in a few states and has been proposed (but not enacted) in US Congress. Another potential solution is a central repository, independent from any physician or health care organization, where manufacturers can donate money that is then distributed for educational purposes.

Some experts have suggested that fraudulent off-label marketing might be prevented through more substantial fines for manufacturers under investigation or other penalties for company managers [53]. Criminal prosecutions of executives are rare [54], but the DOJ has signaled increasing interest in using this approach [53]. While seeking to fortify deterrence through such tactics might address some behaviors, our findings suggest that some common off-label marketing practices may be difficult to control through external regulatory approaches because of their deep-seated nature. Whistleblowers in most of the cases we reviewed reported that private conversations between sales representatives and prescribers were a leading strategy for off-label promotion. The opportunity to prompt and answer physicians' questions about off-label uses, address their individual concerns, and provide a digest of empirical evidence that can be slanted as needed likely makes these conversations a particularly effective form of marketing. The fact that so many of the communications are oral and take place in private offices makes them very difficult for regulators to monitor and sanction. It is impossible to conceive of how anyone other than a company insider or a physician could bring many of these marketing practices to light (indeed, this underlines the distinctive strength of our data source). The move by a few prominent academic medical centers to ban sales representatives from the premises is a bold and powerful one, but it has not, as yet, been followed by many hospitals or physician practices.

Changes in the PhRMA Code are a positive sign that the industry is responsive to public concerns about inappropriate marketing practices. In some news reports, manufacturers have described new corporate cultures that avowedly reject the illegal tactics described in the whistleblower complaints [55]. However, in many of the cases we studied, manufacturers were reported to demonstrate awareness of existing regulations and engage in strategic behaviors to work around them (e.g., by giving employees lectures about the regulatory environment that were understood to be a smokescreen) or to mask their violations of the law (e.g., by encouraging employees to not enter off-label marketing calls in their logs).

Our approach has limitations. First, although the DOJ conducted thorough investigations of each complaint in the study sample, the settled cases concluded without a full trial, which would have included formal fact-finding by a judge or jury. Thus, some allegations may be false and, for nearly all complaints, internal company documents that might have corroborated the complainants' specific reports remained confidential. Second, our analyses were conducted mainly at the complaint level, but nine of the 18 cases involved more than one complaint (the DOJ permits multiple complaints when each brings new information to bear on the case); the clustering of complaints in some cases may have inflated the reported prevalence of certain behaviors. Third, most whistleblowers were US-based sales representatives with a particular field of vision in relation to their companies' off-label marketing practices. It is possible that other behaviors and strategies exist that the whistleblower did not observe and the government investigations did bring to light. Our reliance on the text of the complaints means that we would have missed these. Finally, the complaints were composed to support claims of fraud under certain specific legislation, including the False Claims Act.

### Conclusion

Off-label marketing has been ubiquitous in the health care system and features some behaviors and strategies that may be resistant to external regulatory approaches. Our findings suggest that no regulatory strategy will be complete and effective without physicians themselves serving as a bulwark against off-label promotion. Aside from sales representatives and other company insiders, who play important roles as whistleblowers, physicians are alone in having a full view of many of the most insidious forms of illegal marketing outlined in the complaints we reviewed. As physicians' understanding of these practices and the consequences of inappropriate off-label promotion for public health evolves, so may their enthusiasm for shutting them down.

## Abbreviations

DOJ: US Department of Justice
FDA: US Food and Drug Administration
